# Effects of High Temperature Exposure on the Wingate Test Performance in Male University Students

**DOI:** 10.3390/ijerph20064782

**Published:** 2023-03-08

**Authors:** Víctor Toro-Román, Isaac Prieto-González, Jesús Siquier-Coll, Ignacio Bartolomé, Francisco J. Grijota, Marcos Maynar-Mariño

**Affiliations:** 1 Faculty of Sport Sciences, University of Extremadura, Avenida de la Universidad s/n, 10003 Cáceres, Spain; vtoro@unex.es (V.T.-R.);; 2 SER Research Group, Center of Higher Education Alberta Giménez, Comillas Pontifical University, Costa de Saragossa 16, 07013 Palma Mallorca, Spain; 3 Department of Sport Science, Faculty of Education, Pontifical University of Salamanca, C/Henry Collet, 52-70, 37007 Salamanca, Spain; 4 Faculty of Life and Nature Sciences, University of Nebrija, Campus La Berzosa, Calle del Hostal, Hoyo de Manzanares, 28248 Madrid, Spain

**Keywords:** anaerobic test, power, heat stress, sauna, warm-up

## Abstract

It has been suggested that heat exposure prior to exercise could induce changes in anaerobic exercise. Therefore, the purpose of this study was to observe the effects of high temperature heat exposure prior to an anaerobic test. Twenty-one men (age: 19.76 ± 1.22 years; height: 1.69 ± 0.12 m; weight: 67.89 ± 11.78 kg) voluntarily participated in this investigation. All of them performed two Wingate tests, vertical jump and macronutrient intake control. On the first day, the test was performed under normal environmental conditions. On the second day, it was performed in a similar way, but with previous exposure to heat at high sauna temperatures (15 min; 100 ± 2 °C). There were no differences in the vertical jump and macronutrient intake. However, the results showed an improvement in power (W) (*p* < 0.05), relative power (W/kg) (*p* < 0.01) and revolutions per minute (*p* < 0.05) 10 s after the start of the test. There was also an increase in thigh (*p* < 0.01) and skin temperature (*p* < 0.01) with pre-heat exposure. The results obtained suggest that this pre-exercise protocol could improve power in short and intensive actions.

## 1. Introduction

Acute exposure to heat produces different effects in the human body due to thermoregulation, triggering changes in blood flow, metabolism, the neuromuscular system and body temperature (Tcore). These mechanisms are accentuated at higher temperatures [1].

It has been proposed that warm-up affects performance through a variety of mechanisms. Most of the effects of warm-up have been attributed to temperature-related mechanisms [2]. An increase in muscle temperature could affect performance through a decrease in viscous resistance of muscles and joints [3]. It has also been suggested that changes in performance after warm-up could be due to an increase in oxygen supply to muscles due to increased vasodilation of muscle blood vessels and increased dissociation of oxyhemoglobin [2]. With respect to energy metabolism, elevated muscle temperature enhances aerobic energy production by accelerating reactions associated with oxidative phosphorylation [4]. Furthermore, physical exercise at high temperatures seems to increase the breakdown of muscle glycogen by increasing epinephrine secretion [5,6].

Heart rate (HR) increases caused by the cardiovascular drift phenomenon produced by the increase in Tcore during exercise, inducing an increase in catecholamines. Wingo and Cureton (2006) observed a significant increase in HR after two tests in ambient (22 °C) and hot (42 °C) conditions [7].

Additionally, it has been reported that metabolism undergoes changes at high temperatures as mentioned above. These changes are a consequence of increased blood flow to the periphery for thermoregulation, resulting in reduced muscle blood flow. In parallel, cardiovascular drift causes a decrease in oxygenation of the blood flow. These factors directly affect aerobic metabolism. Thus, the organism uses a metabolic homeostasis mechanism, making greater use of the anaerobic system. Similarly, it increases the rate of adenosine triphosphatase (ATP) utilisation in hot environments, there being a greater hydrolysis of creatine phosphate, accompanied by an elevation in anaerobic glycolysis [6,8].

However, most studies have carried out tests on aerobic performance, with reduced research on tests of an anaerobic character, and it is necessary to know the changes produced by the temperature increase in the neuromuscular system. Racinais et al., (2017) observed that fast isotonic contractions are more influenced by temperature in comparison with slower movements [9]. One of the most used tests to assess anaerobic power is the Wingate test. This test was developed in the 1970s. The test was designed to be simple to perform, without the need for specially trained personal; inexpensive; to be used with commonly used equipment; non-invasive; and to measure muscle performance instead of indirect variables (biochemical or physiological). Test–Retest Reliability coefficients have been reported between 0.89 and 0.98, and are usually higher than 0.94. The coefficients tend to be somewhat higher for average power than for peak power, which may reflect a relatively larger error in the measurement of peak power [10].

Matsuura et al. (2015) suggested that additional research is required to further elucidate the relationship between power production profiles and neuromuscular efficiency under heat stress [11]. It has been theorised that tetanic strength could be enhanced due to increased body temperature. This can be caused by increased binding of contractile proteins [12]. Body temperature, and in particular muscle temperature, optimizes power output during short-term explosive exercise by regulating neuromuscular function, with a strong association between contracting performance and muscle temperature (Tmuscle) [9]. In response, for each degree of variation, performance could increase by 2–5% [13]. In addition, the rate of muscle development could be increased by the higher temperature [14,15]. This is consistent with a higher ATPase activity of myosin heads [16], in combination with the retention of calcium by the sarcoplasmic reticulum [17]. Consequently, the rate of contraction will be faster [13]. The increase in nerve conduction triggered by the increase in catecholamines induced by heat stress should also be noted. Therefore, in anaerobic tests, power production could be enhanced. It has been documented that power is improved in heat stress situations, however, there are limited research studies where the effect of previous exposure to heat on power is observed, and the results are inconclusive [18,19,20,21]. These results could be explained by the fact that the thermal stimulation was not sufficient to produce the mechanisms described above.

Therefore, this research aimed to observe the effect of previous exposure to heat at high temperatures (100 ± 2 °C) on muscle power, heart rate and perception of effort using the Wingate anaerobic test.

## 2. Materials and Methods

### 2.1. Participants

Twenty-one subjects participated in the present study (Table 1). All participants were undergraduate students of Sports Science at the University of Extremadura located in the city of Cáceres (Spain) and lived in the same city.

Participants were recruited through leaflet distribution and voluntary informative talks. All participants were informed about the purpose of the study and signed a consent form. The protocol was approved by the Biomedical Ethics Committee of the University of Extremadura following the guidelines of the Helsinki ethical declaration, (code: 33/2020). Participants completed the International Physical Activity Questionnaire—Short Form Spanish version to assess physical activity levels [22]. A researcher assisted the participants in responding.

For inclusion in the study, participants had to fulfil the following criteria: men with no concurrent diseases that might affect physical performance, who have not suffered muscular or joint injuries or immobilisations in the previous 6 months, have not taken any supplements, drugs or alcohol in the previous month and during the study, are physically active, have not previously performed the Wingate test and have not previously used sauna baths.

### 2.2. Study Design

**Figure 1:**
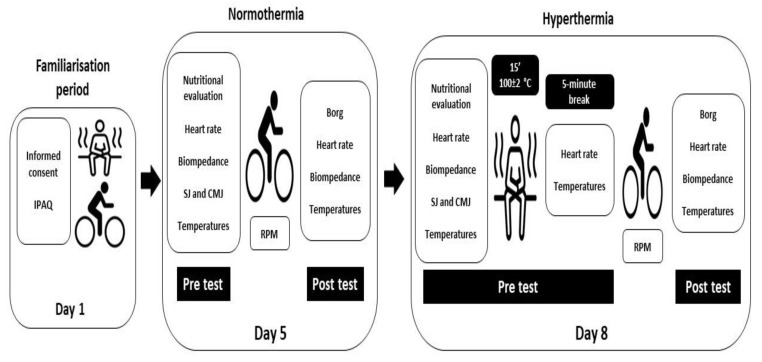
Study design. IPAQ: international physical activity questionnaire; SJ: squat jump; CMJ: countermovement jump; RPM: revolutions per minute.

The investigation was divided into three days: familiarisation period (day 1), performance of the Wingate test under normothermic conditions (day 5) and performance of the Wingate test after exposure to high temperatures (100 ± 2 °C) (day 8). All participants completed the study by carrying out the three-day assessments detailed below. The assessments were performed following the guidelines of previous studies [23,24,25] (Figure 1). All sessions were performed indoors at the same time of day (between 9:30 a.m. and 01:30 p.m.) to minimize the effects of circadian cycles. All tests were performed indoors in the laboratory (22 ± 2 °C, 40–60% relative humidity) [26].

### 2.3. Familiarisation Period and Security Protocol

All participants underwent a familiarisation period to ensure that all subjects were healthy and had no contraindications to participate in the study. During this day the participants performed a Wingate test to become familiarised with the test and to experience the sensations. Likewise, after the Wingate test, the participants went into the sauna at approximately 80 °C for 15 min, and heart rate, blood pressure and body temperature were monitored.

All participants were examined by a physician before, during and after exposure to high temperatures. The medical examination consisted of an electrocardiogram (Sanro BTL-08 SD ECG) and monitoring of blood pressure (Visomat; comfort 20/40) and heart rate (Polar^®^ H10, Kempele, Finland). To monitor temperature, forehead temperature was measured with an infrared thermometer [TAT 5000 “Exergen Temporal Scanner” (Corp., Watertown, MA, USA)] at the beginning, during, and end of exposure to high temperatures. No problems or contraindications were reported during the study.

### 2.4. Nutritional Evaluation

To ensure that all participants had adequate carbohydrate intake to optimally perform the Wingate test [27], all completed a nutritional survey using the MyfitnessPal application [28]. Macronutrients and energy were obtained for subsequent analysis.

### 2.5. Body Composition Measurements

Body composition was measured using electrical bioimpedance (Body Composition Analyzer BF-350, Tanita Corp., Tokyo, Japan). All assessments were performed in the morning, with subjects fasted, barefoot and wearing as little clothing as possible, and following the guidelines in the manufacturer’s manual.

### 2.6. Vertical Jumps

To determine lower limb power, two vertical jump tests were performed: the squat jump (SJ) and countermovement jump (CMJ). An infrared system (OptoJump, MICROGATE, Bolzano, Italy) was used to measure the height and time of flight during the jumps. Two test jumps and two attempts were made for all jump types. There was a 30 s rest between jumps. The best jump was selected for further analysis. Assessments were performed before sauna exposure and the Wingate test. The tests were performed according to Komi and Bosco’s guidelines [29].

To perform SJ, participants started in a squat position with knees bent at approximately 90° and arms on hips. Participants were required to remain in this squat position for 3 s before performing the SJ. For CMJ, subjects started from an upright position with their hands on their hips. Then, they performed a quick downward movement, followed by a jump.

### 2.7. Wingate Test

The Wingate test was used to assess anaerobic power [10]. A Monark 834E cycle ergometer (Crescent AB, Varberg, Sweden) was used. The seat height was adjusted for each participant before the test. The load applied to the cycle ergometer for each participant was calculated following the guidelines of Dotan and Bar-Or (1983) considering that they were sports science students (load = weight × 0.087) [30]. Prior to the test the participants performed a 5 min warm-up at 80–90 revolutions per minute (rpm) interspersed with a 2–3 s sprint every minute without additional load. During the Wingate test the participants had to exercise at maximum rpm for 30 s. Among the researchers in charge of controlling the test, one was in charge of controlling the time, another was in charge of indicating the rpm every 5 s, and another was in charge of recording the rpm indicated by the evaluator. Power was calculated using the formula described by [31]. Heart rate was monitored (Polar^®^ H10, Kempele, Finland). At the end of the test the subjects were asked for their rating of perceived exertion (RPE) with the Borg scale [32]. The scale consisted of 15 points (6 = very very light; 20 = very very hard).

### 2.8. Body Temperature

Body temperature was taken before and after the Wingate test while in normothermic conditions while in hyperthermic conditions, the temperature was taken before and after sauna baths [TAT 5000 “Exergen Temporal Scanner” (Corp., Watertown, MA, USA)]. In addition, the skin was dried with paper after the Wingate test. The temperatures of the forehead and thigh of the dominant leg were taken. After each measurement, the thermometer was cleaned with alcohol and dried with paper.

### 2.9. Heat Exposure

Exposure to the high temperature consisted of a single 15 min session at 100 ± 2 °C and 20% relative humidity (RH) in a Finnish sauna (Harvia C105S Logix Combi Control; 3–15 W; Muurame, Finland). Heart rate was monitored during heat exposure with a pulsometer (Polar^®^ H10, Kempele, Finland). After the heat exposure, the participants rested for 5 min, at room temperature (20–23 °C), before warming up for the Wingate test.

### 2.10. Statistical Analysis

Data were processed using IBM SPSS 25.0 Statistics for Macintosh (IBM Corp., Armonk, NY, USA). Data were expressed as mean ± standard deviation. The normality distribution of the variables was analysed using the Shapiro–Wilk test and the homogeneity of variances using the Levene test. Student’s *t*-test and a two-way ANOVA (group effect + time effect) were used. The percentage change during the Wingate test was calculated every 5 s. A *p* < 0.05 was considered as statistically significant.

## 3. Results

Table 2 displays the macronutrient intake on the different days. There were no statistical differences between days.

Table 3 reflects the results obtained in the SJ and CMJ. There were no differences between the different days of assessment.

Table 4 reports the changes in temperature and weight in the different assessments. There were differences statistically significant between groups in the thigh temperature (*p* < 0.05) and in the different assessments of the forehead (*p* < 0.01). Specifically, there were differences between baseline and post-sauna assessment (*p* < 0.05), as well as between baseline and post-Wingate assessments (*p* < 0.05). There were also differences between post-sauna and post-Wingate ratings (*p* < 0.05).

Table 5 shows the results obtained during the Wingate test in the different assessments. There were significant differences at 5″ in RPM, absolute power and power relative to body weight (*p* < 0.05). There were also significant differences in power at 10″ (*p* < 0.05). However, there were no significant differences in HRmax, RPE and watts from 15″ to the end of the test.

**Figure 2:**
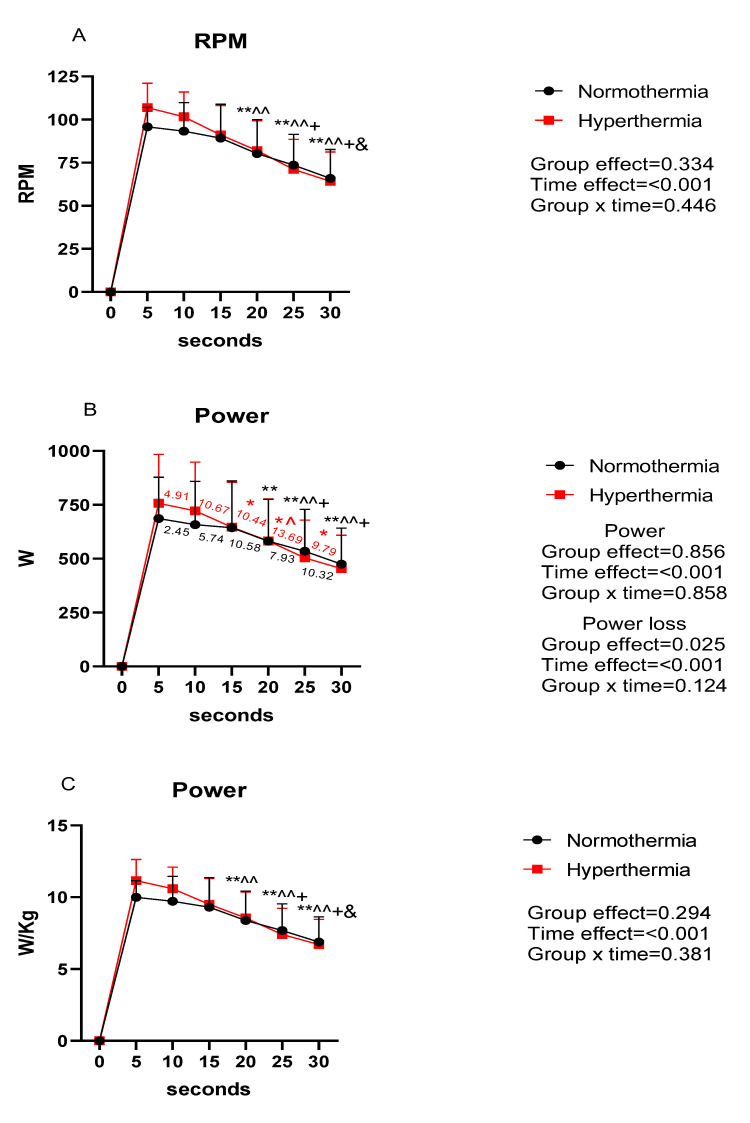
Evolution of RPM and power during the test. (**A**): evolution of RPM throughout the test; (**B**): evolution of absolute power during the test; (**C**): evolution of power values relative to weight throughout the test; RPM: revolution per minutes; red number: power losses of the hyperthermia group with respect to 5″ later; black number: power losses of the hyperthermia group with respect to 5″ later; * *p* < 0.05; ** *p* < 0.01 differences with respect to 5″; ^ *p* < 0.05, ^^ *p* < 0.01 differences with respect to 10″; + *p* < 0.05 differences with respect to 15″; and & *p* < 0.05 differences with respect to 20″.

Figure 2 illustrates the data obtained in RPM, absolute power and power relative to body weight throughout the Wingate test. In addition, the loss of power during the test is also shown. There were significant differences throughout the test in RPM, absolute power, relative power and loss of velocity (*p* < 0.001). Specifically, there were differences with respect to the 5″, 10″, 15″ and 20″ (*p* < 0.05). In terms of between-group differences, there were significant differences in loss of velocity (*p* < 0.05). Specifically, there were differences with respect to loss of velocity at 5″ and 10″ (*p* < 0.05).

## 4. Discussion

This research aimed to observe the effect of previous exposure to heat at high temperatures (100 ± 2 °C) on muscle power, heart rate and perception of effort using the Wingate anaerobic test.

It has been widely supported that HR raises in hot environments. It is due to cardiovascular drift, as discussed. As mentioned above, the increase in HR during exercise is caused by the action of catecholamines, and this mechanism is exacerbated in situations of heat stress. This rise in HR leads to a drop in stroke volume (SV), and therefore cardiovascular drift. However, no changes were obtained in this study. This could be for two reasons: (i) the heat exposure was previous, so the heat stimulus was not maintained, and (ii) due to the duration of the test, as it is more complicated to reach HR_max_ intensities in tests where the time intervals are short (30 s). In aerobic tests to exhaustion, a significant increase in FC_max_ is observed in heat stress. Nevertheless, Kenefick et al. (2009) did not find significant differences in HR_max_ in a maximal incremental aerobic test in normothermia after 15 min of exposure to heat (50 °C) [20]. Thus, the effect of heat stress must be maintained during exercise to cause changes in HR.

In relation to temperature, an increase in thigh temperature (Tt) and skin temperature (Tskin) were documented. A rise in Tskin and Tt have been linked to enhanced fatigue induction during maximal sustained voluntary isometric contractions [33,34,35] and impair aerobic performance. However, no such effect was observed in this study. This could have been because the contractions were isotonic, and the test was anaerobic. Hence, it has been reported that elevated heat stress can lead to a reduction in voluntary muscle activation and loss of force production capacity [9]. However, a recent study reported improvements in 1RM after 12 min of pre-test exposure to 100 °C [36]. Hyperthermic physical exercise results in a higher proportion of recruitment of fast-twitch fibers, with fast twitch fibers being more susceptible to thermal changes compared to slow twitch fibers [6]. Otherwise, muscle contraction speed is influenced by muscle temperature [13], whereas a decrease in Tt can slow down chemical reactions, delay the cross-bridge cycle [37] and decrease actomyosin sensitivity to calcium [38]. Conversely, an increase in Tt could increase the speed of force development of a muscle contraction [14,15], probably related to an exacerbation, due to the temperature of myosin adenosine triphosphatase (ATPase) activation [16], and calcium binding by the sarcoplasmic reticulum [9]. Maximal tetanic force can still be enhanced by raising Tt [36], thereby possibly enhancing the binding of contractile proteins [12]. Within skeletal muscle, an increase in Tcore (e.g., 2–3 °C) elevates contractile speed and modifies the force/frequency ratio [39,40]. Thus, in this study, an increase in power and relative power (W/kg) is observed during the first 10 s, with no differences observed thereafter. It is possible that this increase in force is due to the aforementioned ATPase activity. Furthermore, the duration of the ATP energy system is about 10 s, which is when the effects occur after prior exposure to hyperthermia, so this would support that it is due to the increased ATPase activity. In this line of thought, Falk et al. (1998) reported that pre-heat exposure can improve anaerobic performance [41]. Furthermore, Febbraio (2001) suggested that heat produces a greater reliance on the glycolytic system [2]. However, in this study there was only an increase in performance up to 10 s (alactic anaerobic power). Furthermore, Backx et al. (2000) performed a Wingate test in different environmental conditions of heat and RH (22 °C/30% RH vs. 30 °C/85%RH vs. 40 °C/40%RH) without observing changes [19]. However, other research did find improvements in repeated sprints after exposure to hot water at 40 °C [21]. Chodor et al. (2021) found improvements in repeated sprints in hot conditions (28.5 °C/58% RH vs. 20.5 °C/58.7RH) [42]. Likewise, a study where subjects performed two 30 s sprint tests on a cycle ergometer was followed by comparing the effects of previous immersion in hot water at 40 °C (28.5 °C/58% RH vs. 20.5 °C/58.7% RH) [43]. The authors obtained a higher mean power in the group that had previous exposure to heat. The authors mention that the heat-induced increase in catecholamines causes the interaction of exercise and heat to elicit a further increase in the sympathetic nervous system (SNS) stimulus response [36]. This explanation could be another reason for the increase in power at the beginning of the test in our study. Thus, a raise in body temperature and Tm triggers an improvement in skeletal muscle explosive performance (e.g., sprints and jumps) by improving nerve conduction, conformational changes associated with muscle contraction, and metabolic and contractile function [4].

Concerning fatigue, Iguchi and Shields (2011) applied a passive heat protocol (73 °C) prior to exercise [44]. These authors did not observe immediate changes in fatigue after exercise as we have, where there was no difference in the fatigue index. It should be noted that these authors suggest that the changes occur chronically and not acutely. No significant differences were found in the RPE either. In this respect, Racinais et al. (2019) informed that the increase in “cognitive load” induced by heat exposure could be linked to the feeling of unpleasantness when homeostasis is disturbed [12]. Gaoua et al., 2012 and Racinais et al., 2017) reported that passive hyperthermia may increase the negative effect [45,46], potentially reducing motivation and thus affecting exercise [47,48]. Flouris and Schlader (2015) reported that an elevation of skin temperature could impair performance at the onset of exercise [49]. In contrast, Ely et al. (2009); Périard and Racinais (2015); Périard and Racinais (2015); and Periard and Racinais (2016), reported that exercise is initiated equally at different temperatures, and the initial assumption seems to be inconsistent [50,51,52,53]. In addition, this was not observed in our study, the results being better at the beginning of the test. Thus, it appears that physical performance may be more affected by physiological tension derived from stress. In this regard, Chaâri and Frikha (2022) implemented a 20 min vs. 10 min warm-up protocol in hot conditions (31 °C), finding a decrease in power in a repeated sprint test (RSA) in the 20 min protocol [54]. Therefore, in this study, the protocol was performed for 15 min, and after this, improvements in power were found in the first 10 s.

Finally, this research has several limitations. First, the sample size. Second, a crossover study was not performed, however, the previous jump values inform us that there were no differences between the power of the subjects prior to the application of the protocol. Third, sample size was not calculated. Fourth, sleep hours were not monitored. Lastly, no lactate samples were taken that could have provided more information on energy metabolism in the test. Further research is needed to clarify these results by assessing energy metabolism during the test.

## 5. Conclusions

Exposure to heat at high temperatures (100 °C) prior to exercise induced a temperature increase in the muscle that improved power (W), relative power (W/kg) and RPM in the Wingate test compared to normothermia conditions. Considering the results obtained, this protocol improves anaerobic alactic power and can be implemented in short duration explosive sports (<10 s) just before the competition for possible performance improvement.

Future investigators should verify the safety of the protocol, as well as determine markers of cardiac and other markers of muscle damage.

## Figures and Tables

**Table 1:** Characteristics of the participants.

Parameters	*n* = 21
Age (years)	19.76 ± 1.22
Height (m)	1.69 ± 0.12
Weight (kg)	67.89 ± 11.78
Fat mass (kg)	16.12 ± 3.17
Fat mass (%)	14.84 ± 4.23
Free fat mass (kg)	54.42 ± 8.75
Free fat mass (%)	80.03 ± 6.52
Physical activity (MET-min/week)	5017.07 ± 2420.24

**Table 2:** Macronutrient intake before assessments.

Parameters	Normothermia	Hyperthermia	*p*
Energy (kcal/day)	2054.87 ± 361.21	2165.41 ± 289.21	0.614
Carbohydrates (g/kg/day)	2.68 ± 1.18	2.85 ± 1.67	0.426
Fat (g/kg/day)	1.63 ± 0.71	1.58 ± 0.84	0.714
Proteins (g/kg/day)	1.18 ± 0.19	1.21 ± 0.24	0.589

**Table 3:** Vertical jump tests.

Parameters	Normothermia	Hyperthermia	*p*
SJ (s)	0.714 ± 0.46	0.681 ± 0.49	0.588
SJ (cm)	37.52 ± 9.29	39.36 ± 11.21	0.573
CMJ (s)	0.762 ± 0.43	0.786 ± 0.41	0.840
CMJ (cm)	41.23 ± 9.49	41.18 ± 10.83	0.980

SJ: squat jump; CMJ: countermovement jump.

**Table 4:** Changes in body weight and body temperatures in the different evaluations.

Parameters	Time	Normothermia	Hyperthermia	Group Effect	Time Effect	Group × Time
T skin (°C)	Basal	36.31 ± 0.47	36.29 ± 0.47	0.266	0.000	0.431
	Post sauna	-	37.35 ± 0.66 *^			
	Post test	36.10 ± 0.43	36.18 ± 0.53			
T thigh (°C)	Basal	28.50 ± 2.18	28.47 ± 1.28	0.020	0.000	0.104
	Post sauna	-	36.00 ± 2.11 **^^			
	Post test	28.43 ± 2.44	31.00 ± 3.14 +			
Weight (kg)	Basal	68.93 ± 10.32	69.25 ± 8.19	0.381	0.201	0.415
	Post test	68.54 ± 9.14	68.29 ± 9.76			

T: temperature; * *p* < 0.05; ** *p* < 0.01 basal vs. post sauna; + *p* < 0.05 basal vs. post Wingate; ^ *p* < 0.05; ^^ *p* < 0.01 post sauna vs. post Wingate.

**Table 5:** Wingate test results.

Parameters	Time	Normothermia	Hyperthermia	*p*
HR_min_ (bpm)		75.38 ± 10.27	74.89 ± 10.27	0.884
HR_post sauna_ (bpm)		-	113.63 ± 22.72	-
Hr_max_ (bpm)		181.76 ± 6.73	183.16 ± 9.52	0.304
Load (kg)		5.85 ± 1.31	5.83 ± 1.37	0.963
RPE (point)		17.14 ± 2.12	17.74 ± 1.76	0.390
RPM	5″	95.76 ± 11.23	106.89 ± 14.24	0.038
	10″	93.23 ± 16.58	101.57 ± 14.40	0.072
	15″	89.19 ± 19.74	91.10 ± 17.06	0.746
	20″	80.28 ± 19.64	82.00 ± 17.17	0.772
	25″	73.61 ± 17.83	71.05 ± 17.45	0.649
	30″	65.90 ± 16.76	64.26 ± 16.95	0.760
Power (W)	5″	686.40 ± 191.27	756.85 ± 226.89	0.027
	10″	657.91 ± 200.82	722.12 ± 226.03	0.044
	15″	643.85 ± 217.17	646.27 ± 208.51	0.851
	20″	580.99 ± 194.22	582.45 ± 194.86	0.742
	25″	534.53 ± 194.22	503.85 ± 174.51	0.419
	30″	474.57 ± 167.92	453.49 ± 155.10	0.529
Power (W/kg)	5″	9.99 ± 1.17	11.15 ± 1.48	0.002
	10″	9.72 ± 1.73	10.60 ± 1.50	0.135
	15″	9.31 ± 2.06	9.51 ± 1.78	0.484
	20″	8.38 ± 2.05	8.56 ± 1.79	0.522
	25″	7.68 ± 1.86	7.41 ± 1.82	0.882
	30″	6.88 ± 1.75	6.70 ± 1.76	0.971
Power_max_ (W)		708.96 ± 202.85	761.09 ± 226.47	0.239
Power_mean_ (W)		602.37 ± 192.08	610.84 ± 192.42	0.590
Power_Max_(W/kg)		10.48 ± 1.49	11.78 ± 1.71	0.231
Power_mean_ (W/kg)		8.73 ± 1.68	8.99 ± 1.59	0.622
FI		34.99 ± 11.47	40.89 ± 11.83	0.118

HR: heart rate; RPE: Rating of Perceived Exertion; FI: fatigue index.

## Data Availability

Not applicable.
